# Paediatric Takayasu’s arteritis complicated by thrombotic occlusion of the distal thoracic aorta

**DOI:** 10.1093/icvts/ivab302

**Published:** 2021-11-03

**Authors:** Tomohiro Nakata, Maiko Tachi, Shoichi Suehiro, Teiji Oda

**Affiliations:** Department of Cardiovascular Surgery, Shimane University Faculty of Medicine, Shimane, Japan

**Keywords:** Pediatric Takayasu’s arteritis, Thrombotic occlusion, Mid-aortic syndrome

## Abstract

We present the case of a 1-year-old girl with mid-aortic syndrome due to untreated Takayasu’s arteritis who developed cardiogenic shock. Enhanced computed tomography revealed long-segment occlusion of the distal thoracic aorta. We successfully performed graft interpose (10 mm in diameter) under cardiopulmonary bypass through both median sternotomy and left posterolateral thoracotomy. The thrombus was relatively small and the distal thoracic aorta was narrow over a long segment due to severely thickened intima. Follow-up computed tomography showed widely patent graft without a stenotic region in the abdominal aorta or its branches. The patient discharged ambulatory without major complications.

## INTRODUCTION

Takayasu’s arteritis (TA) is an inflammatory, granulomatous and fibrosing arteritis of the aorta and its major branches [1, 2]. Paediatric TA is a devastating disease with significant morbidity and mortality. However, the diagnosis of paediatric TA might be difficult, partly because the clinical features of TA are non-specific [1]. We encountered a 1-year-old girl with untreated TA who developed cardiogenic shock due to thrombotic occlusion of the distal thoracic aorta.

## CASE REPORT

**Figure 1:**
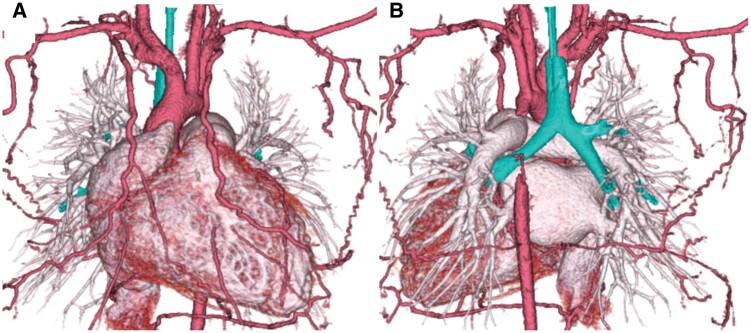
Preoperative computed tomography showing long-segment obstruction from the distal arch to the descending thoracic aorta. (**A**) Anterior view and (**B**) posterior view.

A 1-year-old girl (height, 80 cm; weight, 10 kg) was referred to our institution due to cardiogenic shock. Chest X-ray showed cardiomegaly and severe lung congestion. PaO_2_/FiO_2_ ratio was 127. Echocardiogram revealed reduced ejection fraction and moderate mitral regurgitation without intracardiac or coronary anomalies. Colour Doppler showed obstruction in the arch distal to the left subclavian artery. Enhanced computed tomography (CT) revealed long-segment obstruction from the distal arch to the descending aorta (Fig. 1). Laboratory data showed the following values: aspartate aminotransferase of 9393 IU/l, creatinine of 1.08 mg/dl and brain natriuretic peptide of 12 777 pg/ml.

After admission, the patient was rushed to the operating room. She was put in a supine position with the left chest elevated about 45 degrees. After median sternotomy, she was placed on moderate hypothermic cardiopulmonary bypass. The neck vessels and distal arch were dissected. Then, the descending aorta was dissected through a left posterolateral thoracotomy via the fifth intercostal space. The outer diameter of the descending aorta appeared normal; however, inflammatory adhesion was severe around the obstructive region. After incision of the distal arch, a small thrombus was removed. The intima was severely thickened, and the lumen was severely stenotic for a long distance. Therefore, we abandoned arch reconstruction with native tissue including a sliding arch aortoplasty and chose graft interposition. The narrow descending aorta (6 mm in diameter) was cut obliquely and anastomosed with a 10 mm expanded polytetrafluoroethylene tube through the left thoracotomy (we abandoned direct cannulation of the descending aorta, because of the narrow size). The distal arch was trimmed and anastomosed with the other end of the graft through the median sternotomy. Both proximal and distal stumps were closed directly.

**Figure 2:**
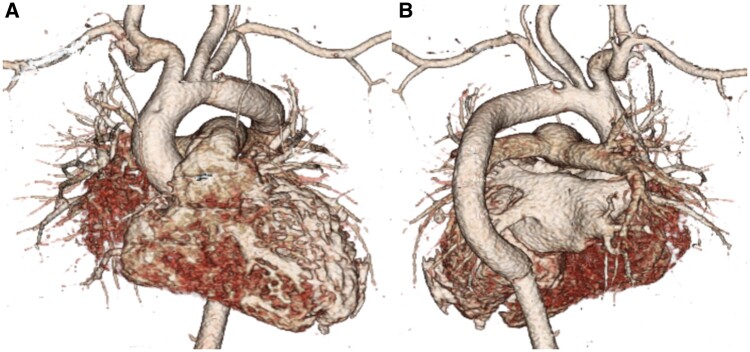
Postoperative computed tomography showing widely patent graft. (**A**) Anterior view and (**B**) right posterior oblique view.

Postoperatively, the patient suffered from acute renal failure and peritoneal dialysis was performed until postoperative Day 18. Histopathologic findings revealed fibrous thickening of the aorta and destruction of the arterial medial elastic fibres by lymphocytes and granulocytes. Therefore, mid-aortic syndrome due to TA was the definitive diagnosis. Enalapril, steroid and aspirin were initiated. Postoperative echocardiography demonstrated good left ventricular function without mitral regurgitation. Postoperative CT showed widely patent graft (Fig. 2). There was no stenotic region in the abdominal aorta or its branches. She was discharged ambulatory without major complications on postoperative Day 59.

## COMMENT

Surgical interventions for TA have been performed to repair atypical aortic coarctation or involvement of abdominal arteries [1, 2]. Most such cases were diagnosed with TA preoperatively and treated with steroids and/or immunosuppressive agents. Moreover, patients with active inflammation at the time of surgery may be at higher risk for vascular complication [2] and controlling the inflammatory process before surgery is preferable.

Mid-aortic syndrome is characterized by a diffuse narrowing of the distal thoracic or abdominal aorta, regardless of aetiology. Percutaneous intervention is employed at many institutions to relieve the stenosis with good results [3]. However, endovascular therapy was considered too risky in this patient, because of the preoperative shock status and the long-segment occlusion.

If collateral circulation restores blood flow to the abdomen in a patient with long-segment aortic occlusion, scheduled revascularization without cardiopulmonary bypass can be performed [4]. Alternatively, if the patient has a bigger physique, a salvage operation such as axillo-external iliac artery bypass can be performed [5]. However, in our patient, we had no choice but to perform revascularization by graft interpose (it was impossible to perform arch reconstruction with native tissue because of long-segment obstruction and the severely thickened intima). Judging from her preoperative heart and lung condition, it appeared dangerous to perform revascularization only through a thoracotomy. Thus, we performed a sternotomy to place her on cardiopulmonary bypass promptly and enable central extracorporeal membrane oxygenation support in case of postoperative cardiac or respiratory failure.

One may argue that extra-anatomic ascending aorta to abdominal aorta bypass might be easily performed via median sternotomy and split midline laparotomy [4]. However, long and curved graft for such a small child should be avoided, because it is difficult to predict how the extra-anatomic graft will be stretched as the child grows, and such a graft would make repeated procedures necessary as the child grows.

In general, some patients with TA will experience a flare up. Patients with lower body mass index have a high risk of poor outcomes [1]. Moreover, disease activity of TA can increase the likelihood of graft revision [2]. Therefore, close monitoring, drug management and long-term follow-up are required. In order to detect new vascular complications early, it is necessary to perform enhanced CT on a regular basis.

**Conflict of interest:** none declared. 

## Reviewer information

Interactive CardioVascular and Thoracic Surgery thanks André Rüffer and the other anonymous reviewers for their contribution to the peer review process of this article.

## References

[1] Fan L , Zhang H, Cai J, Yang L, Liu B, Wei D et al Clinical course and prognostic factors of childhood Takayasu's arteritis: over 15-year comprehensive analysis of 101 patients. Arthritis Res Ther 2019;21:31. 30670069 10.1186/s13075-018-1790-x PMC6341556

[2] Fields CE , Bower TC, Cooper LT, Hoskin T, Noel AA, Panneton JM et al Takayasu’s arteritis: operative results and influence of disease activity. J Vasc Surg 2006;43:64–71. 16414389 10.1016/j.jvs.2005.10.010

[3] Porras D , Stein DR, Ferguson MA, Chaudry G, Alomari A, Vakili K et al Midaortic syndrome: 30 years of experience with medical, endovascular and surgical management. Pediatr Nephrol 2013;28:2023–33. 23775038 10.1007/s00467-013-2514-8 PMC3822337

[4] Coleman DM , Eliason JL, Ohye RG, Stanley JC. Long-segment thoracoabdominal aortic occlusions in childhood. J Vasc Surg 2012;56:482–5. 22560332 10.1016/j.jvs.2012.01.083

[5] Sugawara H , Goto H, Akamatsu D, Hamada Y, Tsuchida K, Yoshida Y et al Midaortic syndrome due to Takayasu arteritis in a child with acute decompensated cardiac failure managed by an emergency axillo-external iliac artery bypass: a follow-up case report of long-term outcomes. Ann Vasc Surg 2020;64:408.e5–e9. 10.1016/j.avsg.2019.09.026 31634602

